# Measurement invariance testing of the PHQ-9 in a multi-ethnic population in Europe: the HELIUS study

**DOI:** 10.1186/s12888-017-1506-9

**Published:** 2017-10-24

**Authors:** Henrike Galenkamp, Karien Stronks, Marieke B. Snijder, Eske M. Derks

**Affiliations:** 1 Department of Public Health and Amsterdam Public Health (APH) research institute, Academic Medical Center, University of Amsterdam, Amsterdam, The Netherlands; 2 Department of Public Health, Academic Medical Center, PO 22660, 1100 DD Amsterdam, The Netherlands; 3 Department of Clinical Epidemiology, Biostatistics and Bioinformatics, Academic Medical Center, University of Amsterdam, Amsterdam, The Netherlands; 4 QIMR Berghofer, Translational Neurogenomics group, Brisbane, Australia; 5 Department of Psychiatry and Amsterdam Public Health (APH) research institute, Academic Medical Center, University of Amsterdam, Amsterdam, The Netherlands

**Keywords:** Measurement invariance, Differential item functioning, Confirmatory factor analysis, PHQ-9, Depressive symptoms, HELIUS study

## Abstract

**Background:**

In Western European countries, the prevalence of depressive symptoms is higher among ethnic minority groups, compared to the host population. We explored whether these inequalities reflect variance in the way depressive symptoms are measured, by investigating whether items of the PHQ-9 measure the same underlying construct in six ethnic groups in the Netherlands.

**Methods:**

A total of 23,182 men and women aged 18–70 of Dutch, South-Asian Surinamese, African Surinamese, Ghanaian, Turkish or Moroccan origin were included in the HELIUS study and had answered to at least one of the PHQ-9 items. We conducted multiple group confirmatory factor analyses (MGCFA), with increasingly stringent model constraints (i.e. assessing Configural, Metric, Strong and Strict measurement invariance (MI)), and regression analysis, to confirm comparability of PHQ-9 items across ethnic groups.

**Results:**

A one-factor model, where all nine items reflect a single underlying construct, showed acceptable model fit and was used for MI testing. In each subsequent step, change in goodness-of-fit measures did not exceed 0.015 (RMSEA) or 0.01 (CFI). Moreover, strict invariance models showed good or acceptable model fit (Men: RMSEA = 0.050; CFI = 0.985; Women: RMSEA = 0.058; CFI = 0.979), indicating between-group equality of item clusters, factor loadings, item thresholds and residual variances. Finally, regression analysis did not indicate potential ethnicity-related differential item functioning (DIF) of the PHQ-9.

**Conclusions:**

This study provides evidence of measurement invariance of the PHQ-9 regarding ethnicity, implying that the observed inequalities in depressive symptoms cannot be attributed to DIF.

## Background

Depression is one of the leading causes of disease burden worldwide, and its prevalence is only expected to increase further [1]. In 2010, major depressive disorder (MDD) accounted globally for 8.2% of years lived with disability. The prevalence of depression differs across demographic groups. For example, meta-analyses showed that individuals with low socioeconomic status (SES) have a higher risk of suffering from a depression, compared to individuals with high SES, while the disease more often has a chronic course in the low SES group [2, 3]. Ethnic inequalities in depression have also been reported in European countries: ethnic minority groups show an increased risk of poor mental health in general, and depression in particular, compared to the host population [4–7].

Increased depression rates among ethnic minority populations are of particular concern, since increases in migration were observed for most western European countries over the past decades [8]. Migrants from non-EU countries face the largest challenges, with regard to socioeconomic conditions and health [8]. For example, in the Netherlands, considerable differences in the prevalence of depression and depressive symptoms were reported for Moroccan and Turkish immigrants, compared to the Dutch population [5, 9]. The 1-month prevalence of depressive disorders was 4% in adults of Dutch ethnic origin, whereas it was 7% in adults of Moroccan origin and even 15% among adults of Turkish origin [5].

A key question that emerges from the ethnic variation in prevalence rates, is whether it reflects actual differences in the occurrence of depression or whether it is due to differences in interpretation or presentation of depressive symptoms in the questionnaire. It is not unlikely that true differences in prevalence rates exist, as depression among ethnic minority groups may be caused by difficulties experienced during or after migration [8, 10, 11]. Perceived ethnic discrimination, for instance, has been shown to contribute to depressive symptoms among ethnic minority groups [9]. Also, the higher prevalence of physical health problems among ethnic minority groups, compared with those of Dutch origin [12], may contribute to increased levels of depression among the minority groups. However, after accounting for differences in SES, perceived ethnic discrimination or in physical disorders and limitations, ethnic differences in depression rates are still observed [7, 9, 13]. It is important to explore the possibility that the interpretation or presentation of depressive symptoms, as assessed by a questionnaire, differ by ethnic background. The current study aims to explore whether differential item functioning may be an explanation for the observed ethnic variation in depression rates.

Differential item functioning (DIF) may occur when people from different ethnic groups report to questions about their mental health in a different way. Depressive symptoms – such as feelings of sadness or disappointment – occur in all cultures and ethnic groups [14]. However, the way they are experienced and expressed may differ across cultures. For example, Chinese people may report feelings of boredom, pain or fatigue, rather than sadness [14]. Non-Western populations in general are often claimed to ‘somatize’ their mood disturbances [15], although other studies have shown that somatizing is rather a global tendency [16–18].

In order to draw conclusions regarding ethnic differences in the prevalence of depressive symptoms, one should verify whether items of a depression questionnaire measure the same concept in all groups, i.e. confirm that the questionnaire is measurement invariant. Measurement invariance implies that individuals’ characteristics which are not part of the construct of interest, such as gender or ethnicity, do not affect individual item scores, other than via the construct of depression [19, 20]. If the assumption of measurement invariance is violated, this implies that the items function differently across ethnic groups. For example, if two individuals of Turkish and Dutch ethnic origin who have a similar level of underlying depression are asked whether they have experienced fatigue, they should have the same probability of responding ‘more than half of the days’. If the expression of fatigue as a symptom of depression is more common for a Turkish individual, this may influence his total depressive symptom score, despite an equal underlying level of depression. Since DIF in one or multiple items would affect the extent to which these items correlate with the remaining items, establishing measurement invariance warrants a systematic analysis of the correlational patterns across items.

Following DSM-5 guidelines, a diagnosis of depression is based on the experience of at least five out of nine symptoms of depression in the last two weeks. We use the PHQ-9 (Patient Health Questionnaire) to assess depressive symptoms in this study, which has the advantage that the items precisely reflect these nine DSM-5 symptoms [21]: depressed mood, anhedonia, trouble sleeping, feeling tired, change in appetite, guilt or worthlessness, trouble concentrating, feeling slowed down or restless, or suicidal thoughts. The PHQ-9 is a well-known and often used measure of depressive symptoms and can be used to assess (significant) depressed mood [22], or as a continuous measure with scores ranging from 0 to 27 [21, 23].

Teresi et al. performed a review on DIF studies in depression measures, which were mainly - but not exclusively – focused on the Center for Epidemiological Studies Depression Scale (CES-D). They found that several items of these scales showed DIF with regard to demographic characteristics, such as age, gender and ethnicity [24]. However, findings on ethnicity-related DIF were inconsistent and none of the reviewed studies examined DIF across ethnic groups in Europe. More recently, Hirsch et al. studied measurement invariance of the PHQ-9 in a selection of about 350 primary care patients with at least one chronic disease. They compared Russian immigrants living in Germany with native-born Russians living in Russia and native-born Germans living in Germany, and concluded that the PHQ-9 measured the level of depressive symptoms in a similar way in these groups [25]. Baas et al. compared about 300 patients of Dutch Surinamese ethnic origin with patients of Dutch origin. They found that in women the PHQ-9 was measurement invariant for ethnicity, but in men, it was only partially measurement invariant [26].

There is a need for further research on whether the PHQ-9 measures depressive symptoms in a similar way across ethnic groups in the general population. Apart from the fact that they compared only two ethnic groups, the two studies mentioned above included patients with a high risk of depression [26], or with at least one chronic disease [25]. This does not provide evidence on whether the PHQ-9 assesses depressive symptoms similarly across ethnic groups in the general population, which is the usual approach to obtain prevalence rates in the population. The current study aimed to address this need, by examining ethnic-related measurement invariance of the PHQ-9, using data from the Dutch HELIUS study. Various ethnic groups, representative for current migrant groups in Europe (Turkish, Moroccan, South-Asian Surinamese, African Surinamese and Ghanaian origin), were included. This epidemiological study included large random samples of these five groups and a comparison group of Dutch ethnic origin (~24,000 in total and 2500–4600 per group), drawn from the general population of Amsterdam. Measurement invariance regarding ethnicity were assessed separately for men and women, since there is a consistent gender difference in the prevalence of depressive symptoms [27, 28].

## Methods

### Sample

The aim and design of the HELIUS (HEalthy LIfe in an Urban Setting) study have been described in detail elsewhere [29, 30]. In brief, the HELIUS study is a multi-ethnic cohort study conducted in Amsterdam, the Netherlands. Subjects were randomly, stratified by ethnicity, selected from the Amsterdam municipality register, and were sent an invitation letter (and a reminder after 2 weeks) by mail. We were able to contact 55% of those invited (55% among Dutch, 62% among Surinamese, 57% among Ghanaians, 46% among Turks, 48% among Moroccans), either by response card or after a home visit by an ethnically-matched interviewer. Of those, 50% agreed to participate (participation rate; 60% among Dutch, 51% among Surinamese, 61% among Ghanaians, 41% among Turks, 43% among Moroccans). Therefore, the overall response rate was 28% with some variations across ethnic groups. After a positive response, participants received a confirmation letter of the appointment for the physical examination, including a digital or paper version of the questionnaire (depending on the preference of the subject). Participants who were unable to complete the questionnaire themselves were offered assistance from a trained ethnically-matched interviewer. The Medical Ethics Committee of the Academic Medical Center (AMC) approved the study protocols. Written informed consent was obtained from all participants involved in the study.

Of the 23,942 participants who filled in the HELIUS questionnaire, we excluded 586 respondents who did not belong to the six largest ethnic groups and an additional 174 respondents who did not fill in any of the PHQ-9 items. Excluded respondents due to missing data were most often of Ghanaian origin, and more often had low or unknown education level, compared to included respondents. All respondents who missed some but not all items were retained in the measurement invariance analyses (*n* = 463), but some were excluded when computing PHQ-9 sum scores (for details we refer to the Measurements section). The majority of those missed only one item (*n* = 396), while the mean number of completed items ranged from 7.5–8 across ethnic groups.

The final sample consisted of 23,182 respondents of Dutch origin (*n* = 4635), South-Asian Surinamese origin (*n* = 3355), African Surinamese origin (*n* = 4428), Ghanaian origin (*n* = 2444), Turkish origin (*n* = 4028) and Moroccan origin (*n* = 4292).

### Measurements

The Dutch version of the PHQ-9 was included in the HELIUS questionnaire [21]. All nine items have four response categories: 0 “not at all”, 1 “on several days”, 2 “on more than half of the days” and 3 “nearly every day”. Total sum scores range from 0 to 27. A participant was considered to have *depressed mood* when having a sum score greater than 9 and *significant depressed mood* when one or both of items 1 and 2 were answered with at least ‘more than half of the days’, and at least 5 of the 9 items were answered ‘more than half of the days’ or ‘nearly every day’. The final item (suicidal ideation) already counted if answered with ‘several days’ [22]. Only for calculating the sum score we replaced missing item scores, or excluded some individuals with more than one missing item. If one of the items was missing, we replaced it by the mean score of the other items and the sum score was calculated as usual. If more than one item was missing, the sum score was not calculated (missing). In subsequent measurement invariance analyses, participants with missing items were all included while missing items were not replaced.

Item 8 of the PHQ-9 originally contained two questions combined in a single item (“Moving or speaking so slowly that other people could have noticed? Or the opposite — being so fidgety or restless that you have been moving around a lot more than usual”), which appeared very difficult to answer when we pre-tested the HELIUS questionnaire. Therefore, in the HELIUS questionnaire, item 8 is divided into 2 items. For all analyses, these items were first combined into a single item, to make this item resemble the one from the original instrument. In all 9 items, we collapsed adjacent response categories in case they contained <5% of the sample, to ensure that endorsement rates were high enough for measurement invariance analyses. This resulted in one dichotomous item (item 9), four items with three categories (items 2, 6, 7 and 8) and four items with four categories (items 1, 3, 4 and 5) (Table 1).

**Table 1:** Item responses (%) of the PHQ-9^a^

In the past 2 weeks, how often have you had the following problems?	Response categories	Dutch	South-Asian Surinamese	African Surinamese	Ghanaians	Turks	Moroccans
1. Little interest or pleasure in doing things	Never	58.7	46.4	53.5	63.1	35.7	39.1
	On several days	35.9	38.0	36.1	24.8	44.1	43.4
	On more than half of the days	3.6	6.8	5.3	8.1	9.9	9.4
	Nearly every day	1.8	8.8	5.1	3.9	10.3	8.1
2. Feeling down, depressed, or hopeless	Never	72.0	61.5	71.8	75.7	52.7	59.2
	On several days	24.4	26.2	21.3	16.2	31.2	28.0
	**On more than half of the days**	**2.5**	**6.0**	**3.8**	**5.7**	**8.5**	**7.2**
	**Nearly every day**	**1.1**	**6.2**	**3.1**	**2.5**	**7.6**	**5.7**
3. Trouble falling or staying asleep, or sleeping too much	Never	46.9	45.8	53.9	67.6	43.2	43.7
	On several days	39.1	31.5	30.3	20.5	29.7	31.7
	On more than half of the days	8.1	8.6	5.6	7.0	11.5	11.1
	Nearly every day	5.8	14.1	10.2	4.8	15.5	13.5
4. Feeling tired or having little energy	Never	37.3	33.0	44.2	52.9	26.2	29.2
	On several days	49.4	42.3	40.6	30.7	40.4	42.0
	On more than half of the days	8.5	10.5	7.2	11.3	14.8	13.3
	Nearly every day	4.9	14.2	8.0	5.2	18.6	15.5
5. Poor appetite or overeating	Never	72.8	56.9	67.9	71.9	52.0	49.3
	On several days	21.5	28.3	22.8	18.8	28.0	32.1
	On more than half of the days	3.6	6.7	4.9	6.6	10.8	10.5
	Nearly every day	2.2	8.1	4.5	2.8	9.1	8.1
6. Feeling bad about yourself or feeling like a failure or like you’ve let yourself or your family down	Never	76.3	74.1	79.2	84.2	71.5	73.6
	On several days	19.4	15.9	15.2	10.3	16.8	16.8
	**On more than half of the days**	**2.7**	**4.5**	**2.9**	**3.0**	**6.0**	**5.0**
	**Nearly every day**	**1.6**	**5.5**	**2.7**	**2.5**	**5.6**	**4.6**
7. Trouble concentrating on things, like reading the newspaper or watching television	Never	72.7	70.7	77.5	78.7	65.6	64.6
	On several days	21.1	17.6	15.6	14.5	19.7	21.1
	**On more than half of the days**	**3.7**	**4.8**	**3.0**	**4.2**	**6.9**	**6.6**
	**Nearly every day**	**2.5**	**6.9**	**4.0**	**2.6**	**7.8**	**7.6**
8. Moving or speaking so slowly that other people might notice, orBeing so fidgety or restless that you move around more than usual (2 items in HELIUS)	Never	86.2	78.9	86.2	84.8	71.3	75.3
	On several days	11.2	13.6	10.0	10.2	16.9	17.2
	**On more than half of the days**	**1.7**	**3.5**	**2.0**	**3.4**	**5.9**	**3.8**
	**Nearly every day**	**0.9**	**4.0**	**1.9**	**1.6**	**5.8**	**3.6**
9. Thinking that you’d be better off dead, or thinking about hurting yourself in some way	Never	94.7	87.5	93.5	95.4	90.3	91.8
	**On several days**	**4.4**	**8.6**	**5.1**	**3.0**	**6.6**	**5.5**
	**On more than half of the days**	**0.7**	**1.6**	**0.7**	**0.9**	**1.5**	**1.2**
	**Nearly every day**	**0.2**	**2.2**	**0.7**	**0.7**	**1.5**	**1.5**

Bold printed categories are collapsed in the analyses because of prevalence lower than 5% in the total sample

^a^Kroenke K, Spitzer RL, Williams JBW: The PHQ-9. Journal of General Internal Medicine 2001, 16(9):606–613

Ethnicity was defined according to the country of birth of the participants as well as that of their parents [31]. Specifically, a participant was considered of non-Dutch ethnicity if either of the following criteria was fulfilled: (1) born outside the Netherlands and at least one parent born outside the Netherlands (i.e., first generation); or (2) born in the Netherlands, but both parents born outside the Netherlands (i.e., second generation). In addition, as the Surinamese population consists of different ethnic groups which cannot be distinguished from each other on the basis of country of birth, self-reported ethnicity was used to determine Surinamese subgroups (either African or South-Asian origin). In order to be sure that the respondents report their geographic origin, rather than the group they feel belonging to, the question on self-identification was phrased in objective terms [31].

Overall, there were three different modes of questionnaire completion: internet (43%) or paper version (31%), or paper version with interviewer assistance (26%). Participants in the Dutch and in both Surinamese groups completed the questions in Dutch. Of the Ghanaian and Turkish subsamples, 78 and 32%, respectively, completed the questions in English or Turkish. Of the Moroccan subsample, about 33% were assisted by an interviewer who filled in the questionnaire in Dutch, but who often spoke Moroccan Arabic or Berber with the respondent. Unfortunately, no detailed information was available on the language of the interview of Moroccan respondents. Sensitivity analyses were performed to examine if the PHQ-9 was measurement invariant regarding language (English vs. Dutch and Turkish vs. Dutch) and interview mode (internet, paper, or interview).

### Statistical analysis

#### Multiple group confirmatory factor analysis (MGCFA)

Multiple group confirmatory factor analysis (MGCFA) was applied to investigate measurement invariance, because it enables the assessment of measurement invariance at different hierarchic levels, and in multiple groups at the same time [20]. In all analyses, ethnic minority groups were compared with the Dutch ethnic origin reference group.

MGCFA is a special case of confirmatory factor analysis (CFA), which requires a prespecified measurement model to be tested. Several studies assume or have shown that the PHQ-9 is unidimensional, indicating that all items measure the symptoms of a single underlying construct (depression) [21, 26, 32, 33]. However, others could not replicate its unidimensional structure [34–37], and provided evidence for a somatic and a non-somatic component, for instance [37]. To improve model fit, previous researchers have added residual covariances [32, 34] or excluded one ore more items [34, 35]. Since there is inconsistency in the best fitting factor model, we first verified the unidimensionality of the PHQ-9, by comparing the fit of three models: 1) a one-factor model, 2) a two-factor model with items 3,4,5,7,8 loading on factor 1 (somatic) and items 1,2,6,9 loading on factor 2 (non-somatic), and 3) a two-factor model based on exploratory factor analysis (EFA). The best of these three models was used as the baseline model for subsequent measurement invariance tests.

#### Testing of measurement invariance

Four hierarchic levels of measurement invariance were tested [20]. Each level implies that more constraints are added to the model (i.e. parameters are equally estimated across groups), with the fit of the model with more constraints being compared to the fit of the less constrained model (i.e. for the non-reference group these parameters were set free). If the more constrained model does not fit significantly worse in comparison with the model that has fewer constraints, this indicates measurement invariance at the tested level.

At the least stringent level, *configural* invariance indicates that the clustering of items and the factors that they represent is similar across groups. This was investigated by evaluating model fit of the baseline model separately for all ethnic groups. *Metric* invariance entails the similarity of factor loadings, and was tested by comparing a model that constrained all factor loadings to be equal across groups, with a configural model where factor loadings were freely estimated across groups. If metric invariance holds, the items load on the latent construct to the same extent for all groups. *Strong* (or scalar) invariance additionally entails the equality of item thresholds. If strong invariance holds this is evidence that there is no additive response bias, indicating that item responses are not systematically higher or lower in one group compared with the other group(s). Finally, *strict* invariance is the most stringent level and reflects that the residual variances, or error terms, of each item are similar across groups.

For all MGCFA analyses we applied Weighted Least Squares Means and Variance adjusted (WLSMV) estimation with theta parameterization in Mplus version 7.4 for statistical analysis with latent variables [38], in which the items were treated as ordinal variables [39]. For each successive step of MI testing, we applied the parameterization described in the Mplus manual [38].

#### Assessment of goodness-of-fit

Goodness-of-fit statistics were estimated for each model and standard criteria were used to evaluate them. The χ^2^ statistic indicates the discrepancy between the covariance matrix of the observed data and the one that is predicted by the factor model. This statistic is sensitive to sample size and often rejects a good fitting model [40, 41]. Therefore, and because it is recommended to use several indices simultaneously [42], we additionally evaluated RMSEA (Root Mean Square Error of Approximation) and CFI (Comparative Fit Index) values which are less sensitive to sample size [43]. A better model fit is indicated by a low RMSEA value and a high CFI value. RMSEA values lower than 0.08 or 0.05 indicate acceptable and good model fit, respectively. CFI values higher than 0.95 and 0.97 indicate acceptable and good model fit, respectively [44].

Differences between successive measurement invariance models were tested using the DIFFtest procedure in Mplus. Similarly to χ^2^, the DIFFtest is influenced to a large extent by sample size, and thus often rejects good fitting models [41]. Therefore, we also evaluated ΔCFI and ΔRMSEA between the more and less constrained models. Only a few simulation studies have reported cut-offs that indicate significant measurement non-invariance, and none of those examined more than two groups [40, 41, 45]. We decided to apply the most conservative cut-offs. Declines in CFI larger than 0.01 and increases in RMSEA larger than 0.015 indicated a significant worsening of fit [40, 41].

#### Impact of DIF on demographic health inequalities

With MGCFA we tested whether differences in overall factor structure were present. This method may be less powerful to detect DIF of individual items, because all item parameters are constrained across groups at the same time. We therefore performed additional tests which were targeted at individual items to explore more subtle levels of DIF which may remain undetected by the MGCFA approach. In case significant DIF at the item level was found, we examined the impact that adjustment for this DIF had on the magnitude of inequalities in depressive symptoms.

First, we conducted regression analysis to detect significant DIF at the item level. To that end, we first saved individual factor scores from each strict invariance model. With logistic regression, we predicted each dichotomized item score with the corresponding factor score and saved the residuals. The residuals represent the variation in item scores not explained by the underlying factor. Subsequently, we performed linear regression with the residuals as the dependent variable, and ethnicity and ethnicity*factor score as independent variables. This was done to conduct one overall test for uniform DIF (analogous to strong invariance) and non-uniform DIF (analogous to metric MI), respectively [46]. The explained variance (R^2^) of this model represents the predictive value of ethnicity for the item score, over and above the predictive value of the underlying factor, and was interpreted as indicative of DIF. Items with an R^2^ of 2% or higher and significant regression coefficients for the predictors ethnicity or ethnicity*factor (*p*-value below 0.05) were selected as items with DIF [46].

Second, if DIF in any of the items was found, we returned to the MGCFA analysis and estimated the impact of adjusting for this DIF. We aimed to compare ethnic inequalities in factor scores, from models that did and did not adjust for DIF. Factor scores from the previously described strict invariance models were regarded as unadjusted for DIF. Adjustment for DIF was done by adapting the strict invariance model so that for items with DIF all threshold constraints across groups were set free. Using means and variances of unadjusted and adjusted factor scores, we estimated two sets of standardized mean differences (Cohen’s d) across ethnic groups. We evaluated whether 95% confidence intervals around d’s unadjusted for DIF and adjusted for DIF showed overlap, which would indicate that the statistically significant DIF that was observed had low impact on the magnitude of demographic health inequalities. Cohen’s d was calculated using the pooled sd as the denominator; conventional thresholds were used to interpret effect sizes as small (d = 0.2), medium (d = 0.5) and large (d = 0.8) [47].

## Results

### Sample characteristics

Table 2 shows the demographic characteristics and distribution of the PHQ-9 in each ethnic group, and by gender. In both genders, PHQ-9 sum scores were highest among respondents with Turkish ethnic origin and lowest among the group with Ghanaian ethnic origin. A similar pattern of ethnic differences emerged for the prevalence of (significant) depressed mood.

**Table 2:** Sample characteristics by ethnicity

		Dutch	South-Asian Surinamese	African Surinamese	Ghanaians	Turks	Moroccans
N		4635	3355	4428	2444	4028	4292
Mean age (sd)		46.2 (14.0)	46.5 (13.2)	39.9 (12.5)	39.7 (13.0)	44.2 (11.5)	43.8 (13.4)
Female gender (%)		54.1	53.6	59.6	61.4	54.9	62.0
PHQ-9 sumscore (Median [Interquartile Range])	Men	2 [4]	3 [6]	2 [4]	1 [4]	4 [7]	3 [7]
	Women	3 [5]	4 [7]	3 [5]	2 [6]	5 [8]	5 [7]
Depressed mood (%)	Men	5.8	14.0	6.4	7.2	18.8	18.2
	Women	8.5	22.7	13.4	9.9	26.4	22.0
Significant depressed mood (%)	Men	2.6	7.7	3.1	4.1	10.8	10.4
	Women	3.1	11.8	5.9	4.4	15.2	11.4

### Measurement invariance analyses

Three different factor models were compared, to obtain an adequate baseline model for further analysis (Table 3): a one factor model, a two-factor model based on the literature, and a two-factor model based on EFA. The EFA two-factor model was slightly different, and had better fit, compared with the two-factor model that was examined in previous studies. Although the two-factor models generally showed better fit as compared to the one-factor model, we decided to continue with the one-factor model for two reasons. First, in both models the two factors showed a high correlation, indicating that they reflect two largely overlapping constructs. Second, the one-factor model had good model fit according to CFI, and also adequate model fit according to RMSEA after residual covariances (between items 1 and 2, items 3 and 4 and items 7 and 8) were added to the model. The fit of this one-factor model is shown for each ethnic group and gender in Table 4. Model fit was better in men as compared to women, but in all groups RMSEA and CFI values were indicative of acceptable or good model fit.

**Table 3:** Comparing the model fit of one-factor and two-factor models

	χ^2^ (df)	RMSEA	CFI	Factor correlation
One factor				
Model	4398.963 (27)*	0.084 (0.082–0.086)	0.978	
With residual correlation items 1 and 2	3457.229 (26)*	0.075 (0.073–0.078)	0.983	
With residual correlation items 1&2, 3&4	2798.221 (25)*	0.069 (0.067–0.071)	0.986	
With residual correlation items 1&2, 3&4, 7&8	2220.704 (24)*	0.063 (0.061–0.065)	0.989	
Two factors^a^ (somatic vs non-somatic)				
Model	3304.548 (26)*	0.074 (0.072–0.076)	0.984	0.924
With residual correlation items 1 and 2	3130.413 (25)*	0.073 (0.071–0.075)	0.984	0.946
Two factors based on exploratory factor analysis^b^				
Model	2306.120 (25)*	0.063 (0.061–0.065)	0.989	0.871
With residual correlation items 1 and 2	1130.192 (24)*	0.045 (0.042–0.047)	0.994	0.875

*Significant χ^2^ test (*P* < .001)

^a^Items 3,4,5,7 and 8 loading on factor 1 and items 1,2,6 and 9 loading on factor 2

^b^Items 1,3,4 and 5 loading on factor 1, and items 1,2,6,7,8 and 9 loading on factor 2

**Table 4:** Model fit of the baseline one-factor model^a^ in each subgroup

	χ^2^ (df)	RMSEA	CFI
Men (*N* = 9863)	767.158 (24)*	0.056 (0.053–0.059)	0.991
Dutch (*n* = 2128)	213.373 (24)*	0.061 (0.054–0.069)	0.982
South-Asian Surinamese (*n* = 1557)	146.761 (24)*	0.057 (0.049–0.066)	0.992
African Surinamese (*n* = 1790)	122.728 (24)*	0.048 (0.040–0.057)	0.988
Ghanaian (*n* = 944)	83.630 (24)*	0.051 (0.040–0.064)	0.988
Turkish (*n* = 1815)	226.109 (24)*	0.068 (0.060–0.076)	0.991
Moroccan (*n* = 1629)	138.988 (24)*	0.054 (0.046–0.063)	0.994
Women (*N* = 13,319)	1376.496 (24)*	0.065 (0.062–0.068)	0.988
Dutch (*n* = 2507)	263.669 (24)*	0.063 (0.056–0.070)	0.981
South-Asian Surinamese (*n* = 1798)	281.817 (24)*	0.077 (0.069–0.086)	0.987
African Surinamese (*n* = 2638)	152.603 (24)*	0.045 (0.038–0.052)	0.993
Ghanaian (*n* = 1500)	113.997 (24)*	0.050 (0.041–0.059)	0.990
Turkish (*n* = 2213)	358.969 (24)*	0.079 (0.072–0.087)	0.984
Moroccan (*n* = 2663)	352.918 (24)*	0.072 (0.065–0.078)	0.988

^a^One-factor model with three residual correlations, between items 1&2, 3&4, and 7&8

*Significant χ^2^ test (*P* < .001)

Results from the MGCFA are shown in Table 5. Adding constraints for equal factor loadings, item thresholds and residual variances did not lead to significantly reduced model fit, compared to the least constrained (configural) model. The final strict measurement invariance models for both men and women showed adequate model fit (Men: RMSEA = 0.050; CFI = 0.985; Women: RMSEA = 0.058; CFI = 0.979), while ΔRMSEA and ΔCFI for increasingly stringent test of measurement invariance never exceeded the critical values of 0.015 and 0.01, respectively. Since model fit – according to RMSEA - differed more between ethnic groups among women than among men (Table 4), we examined whether this was due to DIF with respect to gender in some but not in other ethnic groups. However, the results showed that this was not the case: items of the PHQ-9 were measurement invariant for gender in all ethnic groups (Table 6).

**Table 5:** Measurement invariance tests regarding ethnicity, by gender

	Model	Free parameters	χ^2^ (df)	RMSEA	CFI	Reference model	ΔRMSEA	ΔCFI	Difftest χ^2^ (df)
Men	1.Configural	198	928.088 (144)*	0.058 (0.054–0.061)	0.990				
	2.Metric	158	1274.214 (184)*	0.060 (0.057–0.063)	0.987	1	+0.002	-0.003	349.622 (40)*
	3.Strong (scalar)	103	1261.862 (239)*	0.051 (0.048–0.054)	0.987	2	-0.009	0	150.676 (55)*
	4.Strict	43	1543.709 (299)*	0.050 (0.048–0.053)	0.985	3	-0.001	-0.002	378.881 (60)*
Women	1.Configural	198	1515.877 (144)*	0.066 (0.063–0.069)	0.987				
	2.Metric	158	2236.731 (184)*	0.071 (0.068–0.074)	0.981	1	+0.005	-0.006	681.499 (40)*
	3.Strong (scalar)	103	2278.780 (239)*	0.062 (0.060–0.064)	0.981	2	-0.009	0	315.266 (55)*
	4.Strict	43	2554.394 (299)*	0.058 (0.056–0.060)	0.979	3	-0.004	-0.002	523.376 (60)*

* Significant χ^2^ test or χ^2^ difference test (*P* < .001) (compared to the reference model)

The additional regression analyses, targeted at individual items, revealed no items with DIF related to ethnicity (Tables 7, 8 and 9). Furthermore, sensitivity analyses confirmed that the PHQ-9 was measurement invariant with regard to language and interview mode (Tables 10 and 11).

## Discussion

Measurement invariance of the PHQ-9 regarding ethnicity was examined in a population-based sample including over 23,000 participants. Our results indicated that the PHQ-9 was measurement invariant across groups with Dutch, South-Asian Surinamese, African Surinamese, Ghanaian, Turkish and Moroccan ethnic origin. As such, the observed ethnic differences in PHQ-9 scores may be attributed to true differences in depressive symptoms, and not to factors related to the measurement of these symptoms.

Our results should be interpreted in view of some limitations. Firstly, non-response to this study may in particular be a concern in those with the poorest mental health, the lowest proficiency of the Dutch language, or in the least acculturated individuals. These factors may influence how the PHQ-9 is responded to, and as such non-response may influence the generalizability of our results. Secondly, this study investigated ethnicity-related DIF for the PHQ-9 and the results can therefore not be generalized to other demographic characteristics or to other depression instruments. For example, Schrier et al. found DIF in five items of the CIDI when comparing respondents with Turkish and Dutch origin in the Netherlands [48]. In addition, in their review Teresi et al. (2008) concluded that several items of depression scales showed DIF with regard to demographic or health characteristics. None of the reviewed studies examined DIF across ethnic groups in Europe, however.

Our selection of statistical approaches and criteria for significance and relevance may be of influence on the conclusions that were drawn. We applied MGCFA, which has been shown to perform well to detect different levels of DIF [49], using model fit parameters that were recommended in previous studies [40, 41, 44, 45]. However, little is known about which criteria should be used when sample sizes are large, or when more than two groups are compared at the same time. We recommend that more research is done in this field, to guide researchers regarding which methods and criteria for significant and relevant DIF should or should not be applied. In the current study the results of both MGCFA analysis and logistic regression analysis pointed in the same direction, which strengthens our conclusion about the absence of ethnicity-related DIF for items of the PHQ-9.

Empirical evidence of measurement invariance is essential for making valid health comparisons across demographic groups. Our results imply that the ethnic inequalities in depressive symptoms, that were observed in our study as well as in other studies [5, 6], reflect true differences, and are not likely the result of measurement bias. Thus, the PHQ-9 can be used to make comparisons regarding the prevalence of (significant) depressed mood in groups with different ethnic background in the Netherlands. The Dutch had the lowest prevalence of 3% for significant depressed mood, and the Turks had the highest rate (11% in men, 15% in women), with the rates for the other ethnic minority groups lying in between. Interestingly, the GBD 2010 data indicate that these ethnic minority groups (except Ghanaians) have lower MDD prevalence in their countries of origin [1], which may suggest that adverse circumstances in the host societies (e.g., ethnic discrimination, acculturative stress) might be at play here.

The pattern of ethnic inequalities in (significant) depressed mood that we observed is somewhat similar to what was found by de Wit et al. who used the CIDI (Composite International Diagnostic Interview) to assess depression. They reported the 1-month prevalence of depressive disorders (MDD or dysthymia) in respondents with Surinamese (1%), Dutch (4%), Moroccan (7%) and Turkish ethnic origin (15%) [5]. The pattern of inequalities – increased prevalence in ethnic minorities, with the lowest rates in Ghanaians and African Surinamese, and higher rates in Turks, Moroccans and South-Asian Surinamese - suggests that migration-related factors may be ethnic-specific, and that ethnic minority groups should not be combined without taking the differences between these groups into account [50]. Future studies could be designed to investigate to what extent genetic vs. cultural variation contributes to these ethnic differences in the prevalence of depression.

To our knowledge, our study is the first to assess ethnicity-related measurement invariance of the PHQ-9 in a population-based sample. Previous studies on ethnicity related DIF included people with at least one chronic disease [25], with HIV [32], or with a high risk of depression [26, 33, 51]. In two studies this was done by administering the full PHQ-9 only if respondents endorsed at least one of the key items, for example anhedonia and depressed mood [33, 51]. In particular the inclusion of high-risk patients provides less information on the ethnic diversity among respondents that do not have a high level of depression but nevertheless might respond differently to the questionnaire. This influences the rates of depression in the general population that are found. Moreover, this study assessed measurement invariance in a variety of ethnic groups that are representative for migrant groups in Europe. In a previous study, Baas et al. (2011) compared two ethnic groups in the Netherlands, both including individuals with a high risk of depression. They found that the item on psychomotor problems (item 8) had a higher factor loading and threshold among Surinamese men, compared to Dutch men. This item originally contains two parts (moving or speaking slowly, or being fidgety and restless), which appeared very difficult to answer when we pre-tested the questionnaire. In the HELIUS questionnaire item 8 was therefore divided into 2 items (see Table 1), and it might be that this adaptation has led to the absence of reporting differences between ethnic groups, whereas they were present in the study by Baas et al.

A strong point of this study is that we were able to additionally study possible DIF due to language and interview mode, given the heterogeneity in our sample regarding these factors. We compared Turks who completed the PHQ-9 in Turkish vs. Dutch, and Ghanaians who completed the PHQ-9 in English vs. Dutch. In addition, we compared groups who completed the questionnaire through the internet, on paper, or with the help of an interviewer. We found that the PHQ-9 was measurement invariant regarding language and interview mode. This result is reassuring and confirms the applicability of the PHQ-9 in different samples and settings.

## Conclusion

With the growing ethnic diversity in European populations there is a need for evidence on the reliability of instruments to study the mental health of ethnic minority groups. The PHQ-9 is often used to measure depressive symptoms in clinical practice or for research purposes. This study provides evidence for measurement invariance of the PHQ-9 in an ethnically diverse sample in the Netherlands. This implies that items of the PHQ-9 function similarly in people with South-Asian Surinamese, African Surinamese, Ghanaian, Turkish and Moroccan ethnic background, as compared to those with Dutch ethnic origin. Moreover, we showed that language (Turkish vs. Dutch in Turks, and English vs. Dutch in Ghanaians) and interview mode (interview, paper, or internet) did not result in measurement bias, indicating that the PHQ-9 can be used in a variety of settings to compare the level of depressive symptoms across ethnic groups. In conclusion, differences in depression scores and rates of depression across ethnic groups are unlikely to be due to assessment bias suggesting that the contribution of other factors such as migration history and migration status should be explored in future studies.

## Appendix

**Table 6:** Measurement invariance tests regarding gender, by ethnic group

	Model	Free parameters	χ^2^ (df)	RMSEA	CFI	Reference model	ΔRMSEA	ΔCFI	Difftest χ^2^ (df)
Dutch	1.Configural	60	511.324 (54)*	0.060 (0.056–0.065)	0.980				
	2.Metric	52	558.816 (62)*	0.059 (0.054–0.063)	0.979	1	-0.001	-0.001	74.071 (8)*
	3.Strong (scalar)	47	541.627 (67)*	0.055 (0.051–0.060)	0.980	2	-0.004	+0.001	9.856 (5)
	4.Strict	35	533.043 (79)*	0.050 (0.046–0.054)	0.981	3	-0.005	+0.001	37.226 (12)*
South-Asian Surinamese	1.Configural	60	531.086 (54)*	0.073 (0.067–0.078)	0.986				
	2.Metric	52	434.464 (62)*	0.060 (0.055–0.065)	0.989	1	-0.013	+0.003	-^a^
	3.Strong (scalar)	47	419.900 (67)*	0.056 (0.051–0.061)	0.990	2	-0.004	+0.001	6.543 (5)
	4.Strict	35	354.010 (79)*	0.046 (0.041–0.050)	0.992	3	-0.010	+0.002	14.017 (12)
African Surinamese	1.Configural	60	376.201 (54)*	0.052 (0.047–0.057)	0.987				
	2.Metric	52	381.536 (62)*	0.048 (0.044–0.053)	0.987	1	-0.004	0	21.671 (8)*
	3.Strong (scalar)	47	384.213 (67)*	0.046 (0.042–0.051)	0.988	2	-0.002	+0.001	16.726 (5)*
	4.Strict	35	416.210 (79)*	0.044 (0.040–0.048)	0.987	3	-0.002	-0.001	62.928 (12)*
Ghanaians	1.Configural	60	201.246 (54)*	0.047 (0.040–0.054)	0.989				
	2.Metric	52	193.254 (62)*	0.042 (0.035–0.048)	0.991	1	-0.005	+0.002	5.186 (8)
	3.Strong (scalar)	47	189.989 (67)*	0.039 (0.032–0.045)	0.991	2	-0.003	0	4.103 (5)
	4.Strict	35	188.652 (79)*	0.034 (0.028–0.040)	0.992	3	-0.005	+0.001	15.131 (12)
Turks	1.Configural	60	746.377 (54)*	0.080 (0.075–0.085)	0.984				
	2.Metric	52	647.073 (62)*	0.068 (0.064–0.073)	0.987	1	-0.012	+0.003	- ^a^
	3.Strong (scalar)	47	646.230 (67)*	0.066 (0.061–0.070)	0.987	2	-0.002	0	24.344 (5)*
	4.Strict	35	532.908 (79)*	0.053 (0.049–0.058)	0.990	3	-0.013	+0.003	16.122 (12)
Moroccans	1.Configural	60	541.256 (54)*	0.065 (0.060–0.070)	0.990				
	2.Metric	52	684.866 (62)*	0.068 (0.064–0.073)	0.987	1	+0.003	-0.003	89.727 (8)*
	3.Strong (scalar)	47	673.573 (67)*	0.065 (0.061–0.069)	0.987	2	-0.003	0	19.178 (5)*
	4.Strict	35	608.601 (79)*	0.056 (0.052–0.060)	0.989	3	-0.009	+0.002	46.250 (12)*

* Significant χ^2^ test or χ^2^ difference test (*P* < .05) (compared to the reference model)

^a^Chi square difference test could not be computed

**Table 7:** Results linear regression analysis on item-specific DIF^a^

	PHQ-9 Item:	1	2	3	4	5	6	7	8	9
		R^2^	R^2^	R^2^	R^2^	R^2^	R^2^	R^2^	R^2^	R^2^
Men:	Ethnicity	0.008	0.005	0.000	0.007	0.004	0.004	0.001	0.001	0.001
	Ethnicity + ethnicity*factor score	0.014	0.009	0.002	0.009	0.006	0.009	0.002	0.004	0.003
Women:	Ethnicity	0.012	0.006	0.001	0.005	0.003	0.002	0.001	0.003	0.002
	Ethnicity + ethnicity*factor score	0.015	0.011	0.003	0.006	0.004	0.003	0.002	0.005	0.003

R^2^ = explained variance

^a^Outcome variable in these linear regression analyses: Residuals that were obtained in logistic regression models with PHQ-9 item scores as outcome variables, and PHQ-9 factor score as the predictor (for all regression coefficients, see Tables 8 and 9)

**Table 8:** Linear regression: Residuals predicted by ethnicity^a^

	Item:	1	2	3	4	5	6	7	8	9
		Unstandardized regression coefficients								
Men:	Dutch (ref)	–	–	–	–	**–**	–	**–**	–	**–**
	South-Asian Surinamese	0.040	0.014	0.021	0.023	**0.110**	0.011	**-0.072**	-0.006	**-0.083**
	African Surinamese	**0.133**	**0.056**	0.017	-0.045	**0.086**	**0.085**	-0.053	0.041	-0.048
	Ghanaians	**0.250**	**0.172**	-0.000	**0.207**	**0.178**	0.050	0.021	0.050	-0.012
	Turks	0.016	-0.006	-0.033	0.033	**0.083**	**-0.054**	-0.051	**0.058**	-0.025
	Moroccans	**0.058**	-0.016	-0.014	-0.019	**0.131**	-0.043	-0.009	-0.024	0.000
	R^2^	0.008	0.005	0.000	0.007	0.004	0.004	0.001	0.001	0.001
Women:	Dutch (ref)	**–**	–	**–**	–	–	–	–	–	**–**
	South-Asian Surinamese	**0.059**	0.051	**-0.070**	0.019	0.010	-0.029	-0.030	0.022	**-0.083**
	African Surinamese	**0.103**	**0.065**	-0.016	0.016	**0.063**	-0.022	-0.007	0.019	**-0.092**
	Ghanaians	**0.354**	**0.242**	**-0.077**	**0.120**	**0.128**	0.054	0.004	**0.113**	-0.011
	Turks	**0.095**	0.046	**-0.080**	**0.162**	**0.109**	**-0.057**	**-0.064**	**0.109**	**-0.069**
	Moroccans	**0.062**	0.015	**-0.073**	**0.114**	**0.105**	**-0.075**	-0.017	0.017	**-0.106**
	R^2^	0.012	0.006	0.001	0.005	0.003	0.002	0.001	0.003	0.002

Bold coefficients were significant at *p* < 0.05, R^2^ = explained variance by ethnicity variable

^a^Residuals were obtained in a logistic regression models with PHQ-9 item scores as outcome variables, and PHQ-9 factor score as the predictor

**Table 9:** Linear regression: Residuals predicted by ethnicity and ethnicity*factor scores^a^

	Item:	1	2	3	4	5	6	7	8	9
		Unstandardized regression coefficients								
Men:	Dutch (ref)									
	South-Asian Surinamese*factor score	0.020	0.003	0.037	**0.036**	**0.039**	0.022	-0.018	-0.032	-0.025
	African Surinamese*factor score	**0.090**	**0.055**	0.035	0.011	0.022	**0.101**	0.002	0.024	-0.001
	Ghanaians*factor score	**0.150**	**0.135**	0.028	**0.093**	**0.079**	**0.069**	**0.058**	**0.081**	**0.060**
	Turks*factor score	-0.022	-0.021	0.031	0.029	0.004	-0.020	-0.012	**0.038**	0.005
	Moroccans* factor score	0.019	-0.002	0.014	-0.003	0.018	-0.027	0.035	-0.007	**0.042**
	R^2^	0.014	0.009	0.002	0.009	0.006	0.009	0.002	0.004	0.003
Women:	Dutch (ref)									
	South-Asian Surinamese*factor score	-0.010	-0.011	0.022	-0.010	-0.028	-0.008	-0.016	-0.006	0.017
	African Surinamese*factor score	0.032	0.025	**0.037**	0.004	0.017	0.005	0.010	-0.013	-0.007
	Ghanaians*factor score	**0.113**	**0.162**	**-0.045**	-0.025	0.040	**0.068**	**0.041**	**0.085**	**0.076**
	Turks*factor score	0.021	0.014	0.028	**0.042**	0.021	-0.024	-0.015	**0.045**	0.023
	Moroccans*factor score	0.016	-0.008	**0.044**	0.020	**0.034**	-0.003	**0.036**	-0.005	0.017
	R^2^	0.015	0.011	0.003	0.006	0.004	0.003	0.002	0.005	0.003

Bold coefficients were significant at *p* < 0.05, b = unstandardized regression coefficients, R^2^ = explained variance by ethnicity and ethnicity*factor score

^a^Residuals were obtained in a logistic regression models with PHQ-9 item scores as outcome variables, and PHQ-9 factor score as the predictor

**Table 10:** Sensitivity analyses: model fit in separate groups regarding interview mode and language

	Free parameters^a^	χ^2^ (df)	RMSEA	CFI
Mode effect, total sample:				
Interviewer (*N* = 6097)	33	668.076*	0.066 (0.062–0.071)	0.988
Internet (*N* = 9904)	33	944.605*	0.062 (0.059–0.066)	0.987
Paper (*N* = 7181)	33	723.689*	0.064 (0.060–0.068)	0.990
Turks only^a^:				
Dutch (*N* = 1874)	33	276.133*	0.075 (0.067–0.083)	0.988
Turkish (*N* = 894)	33	157.153*	0.079 (0.067–0.091)	0.989
Ghanaians only^a^:				
Dutch (*N* = 456)	33	53.541*	0.052 (0.033–0.071)	0.988
English (*N* = 1581)	33	130.249*	0.053 (0.044–0.062)	0.989

^a^Numbers slightly differ from the sample size reported in Table 2 since we excluded those for which the questionnaire language was uncertain

* Significant χ^2^ test or χ^2^ difference test (*P* < .001) (compared to the reference model)

**Table 11:** Sensitivity analyses: measurement invariance analyses regarding interview mode and language

	Model	Free parameters	χ^2^ (df)	RMSEA	CFI	Reference model	ΔRMSEA	ΔCFI	Difftest χ^2^ (df)
Mode effect (Interviewer, Paper, Internet)	1. configural	99	2342.483 (72)*	0.064 (0.062–0.066)	0.989				
	2. metric	83	2627.170(88)*	0.061 (0.059–0.063)	0.987	1	-0.003	-0.002	387.93 (16)*
	3. strong	61	2381.008(110)*	0.052 (0.050–0.054)	0.989	2	-0.009	+0.002	123.571 (22)*
	4. strict	37	2513.875(134)*	0.048 (0.046–0.050)	0.988	3	-0.004	-0.001	444.098 (24)*
Turkish vs. Dutch	1. configural	66	432.981(48)*	0.076 (0.070–0.083)	0.988				
	2. metric	58	558.898(56)*	0.076 (0.070–0.083)	0.986	1	0	-0.002	79.652 (8)*
	3. strong	47	511.195(67)*	0.069 (0.064–0.075)	0.986	2	-0.007	0	45.951 (11)*
	4. strict	35	496.668(79)*	0.062 (0.057–0.067)	0.987	3	-0.007	+0.001	67.021 (12)*
English vs. Dutch	1. configural	66	179.417(48)*	0.052 (0.044–0.060)	0.990				
	2. metric	58	205.994(56)*	0.051 (0.044–0.059)	0.988	1	-0.001	-0.002	31.321 (8)*
	3. strong	47	231.857(67)*	0.049 (0.042–0.056)	0.987	2	-0.002	-0.001	40.683 (11)*
	4. strict	35	251.905(79)*	0.046 (0.040–0.053)	0.986	3	-0.003	-0.001	37.104 (12)*

* Significant χ^2^ test or χ^2^ difference test (*P* < .001) (compared to the reference model)

## Abbreviations

CFA: Confirmatory Factor Analysis
CFI: Comparative Fit Index
CIDI: Composite International Diagnostic Interview
DIF: Differential Item Functioning
MDD: Major Depressive Disorder
MGCFA: Multiple Group Confirmatory Factor Analysis
MI: Measurement Invariance
PHQ-9: Patient Health Questionnaire-9
RMSEA: Root Mean Square Error of Approximation
SES: Socioeconomic status
